# Effect of *Bauhinia monandra* Kurz Leaf Preparations on Embryonic Stages and Adult Snails of *Biomphalaria glabrata* (Say, 1818), *Schistosoma mansoni* Cercariae and Toxicity in *Artemia salina*

**DOI:** 10.3390/molecules27154993

**Published:** 2022-08-05

**Authors:** Thierry Wesley de Albuquerque Aguiar, José Josenildo Batista, Silvio Assis de Oliveira Ferreira, Maíra de Vasconcelos Lima Sampaio, Dewson Rocha Pereira, Magda Rhayanny Assunção Ferreira, Luiz Alberto Lira Soares, Ana Maria Mendonça de Albuquerque Melo, Mônica Camelo Pessoa de Azevedo Albuquerque, André de Lima Aires, Hallysson Douglas Andrade de Araújo, Luana Cassandra Breitenbach Barroso Coelho

**Affiliations:** 1 Centro de Biociências, Departamento de Bioquímica, Universidade Federal de Pernambuco (UFPE), Avenida Prof. Moraes Rego, Cidade Universitária, n 1235, Recife 50670-420, PE, Brazil; 2 Centro de Biociências, Departamento de Biofísica e Radiobiologia, Universidade Federal de Pernambuco (UFPE), Avenida Prof. Moraes Rego, Cidade Universitária, n 1235, Recife 50670-420, PE, Brazil; 3 Centro de Ciências da Saúde, Departamento de Ciências Farmacêuticas, Universidade Federal de Pernambuco (UFPE), Avenida Prof. Arthur de Sá, Cidade Universitária, s/n, Recife 50740-521, PE, Brazil; 4 Centro de Ciências Médicas—Área Acadêmica de Medicina Tropical, Universidade Federal de Pernambuco (UFPE), Avenida Prof. Moraes Rego, Cidade Universitária, n 531-611, Recife 50670-901, PE, Brazil; 5 Laboratório de Imunopatologia Keizo Asami (LIKA), Universidade Federal de Pernambuco (UFPE), Avenida Prof. Moraes Rego, Cidade Universitária, n 1235, Recife 50670-901, PE, Brazil

**Keywords:** embryotoxic effect, schistosomiasis, molluscicide potential, lectin, ecotoxicity

## Abstract

*Biomphalaria glabrata* snails constitute the main vector of schistosomiasis in Brazil, and *Bauhinia monandra* Kurz, the leaves of which contain BmoLL lectin with biocidal action, is a plant widely found on continents in which the disease is endemic. This work describes the composition of *B. monandra* preparations and the effect on embryos and adult snails, their reproduction parameters and hemocytes. We also describe the results of a comet assay after *B. glabrata* exposure to sublethal concentrations of the preparations. Additionally, the effects of the preparations on *S. mansoni* cercariae and environmental monitoring with *Artemia salina* are described. In the chemical evaluation, cinnamic, flavonoid and saponin derivatives were detected in the two preparations assessed, namely the saline extract and the fraction. Both preparations were toxic to embryos in the blastula, gastrula, trochophore, veliger and hippo stages (LC_50_ of 0.042 and 0.0478; 0.0417 and 0.0419; 0.0897 and 0.1582; 0.3734 and 0.0974; 0.397 and 0.0970 mg/mL, respectively) and to adult snails (LC_50_ of 6.6 and 0.87 mg/mL, respectively), which were reproductively affected with decreased egg deposition. In blood cell analysis, characteristic cells for apoptosis, micronucleus and binucleation were detected, while for comet analysis, different degrees of nuclear damage were detected. The fraction was able to cause total mortality of the cercariae and did not present environmental toxicity. Therefore, *B. monandra* preparations are promising in combating schistosomiasis since they can control both the intermediate host and eliminate the infectious agent, besides being safe to the environment.

## 1. Introduction

Plants have been used as sources of raw material since prehistory; it is noticeable that in the last four decades there has been a resurgence of interest in the study and use of plant products [1,2]. According to the World Health Organization (WHO), approximately 85% of the world’s entire population make use of plant extracts to solve problems directly related to primary health care [3]; for example, the molluscicide activity of plants as a prophylactic alternative in the transmission of schistosomiasis [4,5,6].

Schistosomiasis, a parasitic infectious disease caused by parasites of the genus *Schistosoma* spp., affects more than 236 million people in 78 countries and territories worldwide [7]. In Brazil, the disease is endemic in 19 of 27 states, with an estimated 1.5 million infected, with the northeast being the main region affected, putting 25 million people at risk of infection. It is transmitted by mollusks of the genus *Biomphalaria* with *B. glabrata* being the main vector responsible for releasing thousands of cercariae daily into the aquatic environment; in three months, a single mollusk generates approximately 10 million new descendants [8,9,10,11].

The World Health Organization recommends the population control of mollusks with the application of the molluscicide niclosamide (Baylucide, Bayer^®^) only in localities with high prevalence of schistosomiasis, due to its high toxicity to non-target species, such as fish, plants and other organisms present in the aquatic ecosystem [12,13]. Thus, there is an urgent need for investment in research that can contribute to new molluscicide alternatives which are effective, biosafe and present high selectivity. This scenario encouraged our research group to study bioactive natural products in the population control of *B. glabrata* mollusks, their infectious agent (*S. mansoni* cercariae) and the evaluation of environmental ecotoxicity with reference bioindicators [14,15,16].

In this context, plants of the genus *Bauhinia* (Fabaceae family) are widely found on continents where endemic diseases for schistosomes are reported, such as Africa and Asia, as well as Central and South America [17]. In Brazil, *Bauhinia monandra* Kurz [18] is popularly known as “pata-de-vaca” and several of its biotechnological applications have already been reported [19,20]. For example, lectin, a protein that binds to galactose carbohydrate by specific and reversible recognition, was purified to homogeneity from the leaves of *B. monandra* (BmoLL, *B. monandra* leaf lectin) and presented insecticide activity against *Anagasta kuehniella* (Lepidoptera: Pyralidae), *Zabrotes subfasciatus* and *Callosobruchus maculatus* (Coleoptera: Bruchidae) [21,22]. In addition, promising results were observed with endophytic isolated from the leaves of *B. monandra*, which showed fungicidal and bactericidal actions [23,24]. Regarding the toxicity parameter using the *Artemia salina* environmental bioindicator model, one of the most frequently used species for toxicity testing, BmoLL did not affect the survival of nauplii, not even at high concentrations of 250–1000 μg/mL [15]. Different organic extracts of leaves of *B. variegata* showed molluscicidal action against *Lymnaea acuminata* adults, the intermediate host of *Fasciola gigantica*, against which the ethanolic (LC_50_ 14.4 mg/L in 96 h) and the fraction (LC_50_ 20.3 mg/L) were the more effective [25]. Thus, these studies demonstrate that extracts and fractions of species of the genus *Bauhinia* are promising in the control of vectors, including snails, of importance in human and veterinary medicine.

This is the first study to explore the analysis of the composition of the saline extract and fraction of leaves of *B. monandra* and its molluscicidal action on different embryonic and adult stages of *B. glabrata,* as well as reproductive parameters (fecundity and fertility) and cytotoxicity and genotoxicity on hemocytes. Concurrently, the cercaricidal activity on *S. mansoni* (mobility/mortality), the infective evolutionary phase for the definitive host (man), and environmental safety using the bioindicator/non-target organism *Artemia salina* are explored.

## 2. Results and Discussion

### 2.1. Analysis of the Composition of Saline Extract and Fraction of B. monandra Were Assessed

**Figure 1:**
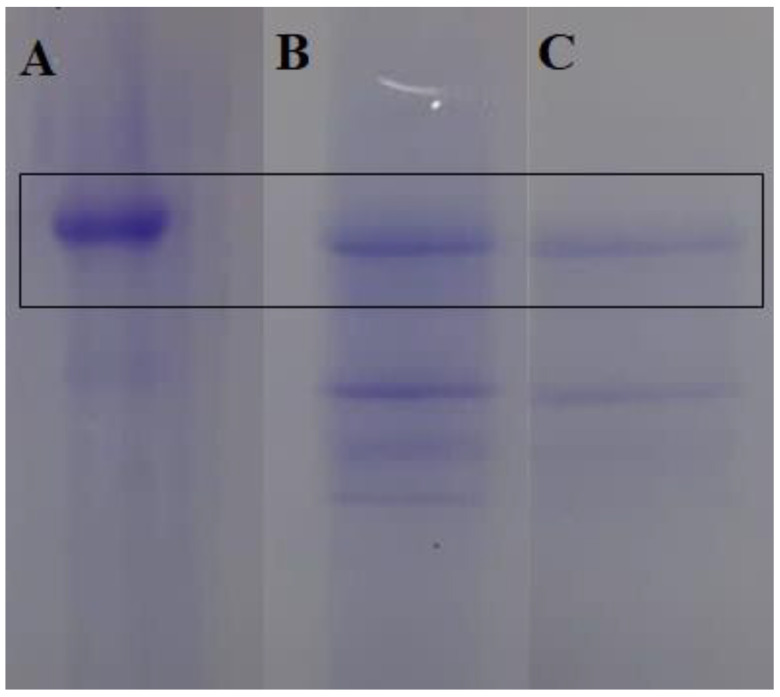
Electrophoresis profile. Application order: (**A**) Lectin BmoLL, (**B**) saline extract and (**C**) fraction.

The saline extract obtained from the suspension in NaCl 0.15 M (0.45% yield—15 g of powder) presented a protein concentration of 19.5 mg/mL, hemagglutinating activity (HA) of 1024 and a specific hemagglutinating activity (SHA) of 52.51. On the other hand, the fraction (0–60%), obtained with ammonium sulfate with 60% saturation, presented a yield of 0.26 mg/100 mL, equivalent to 0.26%, and contained 2.5 mg/mL of protein, 2048 HA and 809.6 SHA, revealing that both preparations contained lectin. While in the electrophoresis profile, BmoLL presented with two bands, one larger (33 KDa) and one smaller (27 KDa) in both preparations (Figure 1), confirming the presence of lectin in the samples.

**Figure 2:**
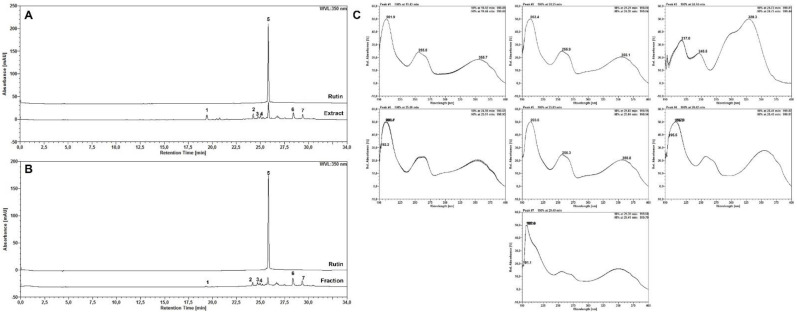
HPLC analysis of *Bauhinia monandra* samples. Chromatogram of the saline extract (**A**) and fraction (**B**); scan spectra (190–400 nm) corresponding to peaks 1 to 7 evidenced in both preparations (**C**). All analyses at 350 nm.

In the TLC analysis, the presence of sugars with reddish-pink bands, saponins with light brown bands, flavonoids with a fluorescent yellow band and cinnamic derivatives with a bluish band were evidenced in the extract, while the fraction presented flavonoids and cinnamic derivatives (same bands but in lower intensity). The following classes of secondary metabolites were not evidenced in the extract and fraction: tannins, alkaloids, anthracenes and coumarins, and saponins were not evidenced in the fraction. Based on these results, the samples were analyzed by HPLC, monitored at 350 nm and the chromatographic profile represented in Figure 2A,B. Several peaks were observed in the HPLC profile with the use of a DAD detector, with five peaks being indicative of the presence of flavonoids (Figure 2C) [peak 1, retention time (rt) = 19.43 min; peak 2, rt = 24.25 min; peak 4, rt = 25 min; peak 5, equivalent to rutin, rt = 25.83 min; peak 7, rt = 29.40 min], one of hydroximic derivative (peak 3, rt = 24.74 min). Using the DAD detector, it was possible to verify that the UV-scan spectra of the compounds observed correspond to the presence of flavonoids in the sample [26], whose absorption maxima were: 255.5/355.7 nm (peak 1); 255.9/355.1 nm (peak 2); 258/354 nm (peak 4); 252/355 nm (peak 5) and 250/270/350 nm (peak 7). The compound evidenced in peak 3, with absorption maxima at 245.5/328.3 nm, corresponds to the presence of a hydroxycinnamic derivative, whose scanning spectrum was similar to the caffeic acid standard analyzed. The presence of rutin was confirmed by injecting the rutin standard, whose peak was observed in the same retention time for the rutin peak in the sample and with the same scanning spectrum. The rutin content for the saline extract and fraction was 0.53 ± 0.0010% and 0.24 ± 0.0033%, respectively. Our results are similar to studies with BmoLL conjugated with quantum dots [20] which showed an HA of 1024 in hemagglutination tests, as well as in studies with extract of *Viola tricolor* [27] and ethanol extract of *Caesalpinia pyramidalis* [28] regarding the presence of this metabolites.

### 2.2. Effect of Preparations on B. glabrata Embryos

The evaluation of embryotoxic activity on embryos of *B. glabrata* snails is an essential aspect for assays of a molluscicide that attests efficiency in the control of *Schistosoma mansoni* [29]. From this perspective, the preparations presented embryotoxicity directly proportional to the concentrations used as observed in Table 1. 

**Figure 3:**
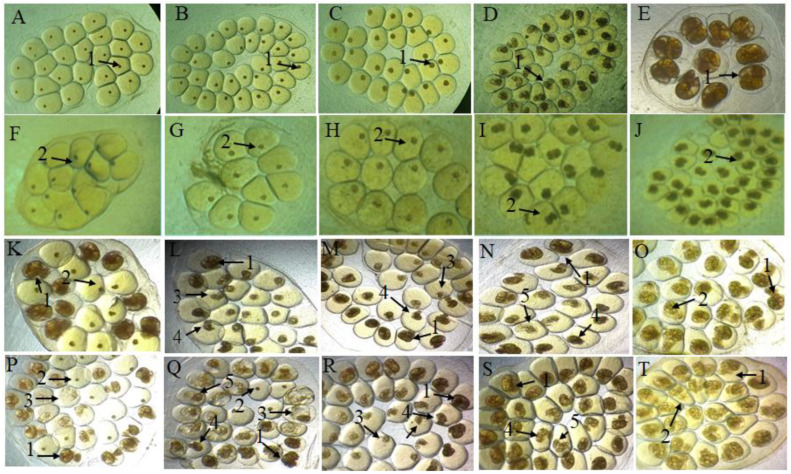
Embryonic stages of B. glabrata and exposure to saline extract and fraction for 24 h. Negative control in blastula stages (**A**); gastrula (**B**); trochophore (**C**); veliger (**D**) and hippo stage (**E**); embryos exposed to the positive control niclosamide, 1.0 µg/mL, (**F**–**J**); embryos exposed to saline extract at different concentrations (**K**–**O**); embryos exposed to the fraction at different concentrations (**P**–**T**). 1: Live embryo; 2: dead embryo; 3: hydropic embryo; 4: embryo with developmental delay; 5: malformation of the shell. All images with 40× magnification.

The embryos exposed to dechlorinated filtered water (negative control) showed a low unviability rate (Figure 3A–E), while the niclosamide (positive control) showed 100% mortality (Figure 3 F–J). The saline extract—at the lowest concentrations (0.0125 to 0.1 mg/mL)—showed unviability percentages of 25.3% to 87.7% and 14% to 71.3% for the stages of blastula and gastrula, respectively, while the fraction presented unviability of 13.3% to 65.3% and 4% to 88.6%, respectively, at the same concentrations. While in the trochophore, veliger and hippo stage, the extract presented—at concentrations of 0.1 to 0.4 mg/mL—non-viability ranging from 63.0%, 23.6% and 16.6% to 90.3%, 59.3% and 51.6%. However, when exposed to the fraction, the embryos in these stages presented greater sensitivity, especially in the concentration of 0.4, which revealed 95.7%, 92.7% and 75.3% unviability. In both preparations, when evaluated for the same stages, they showed 100% unviability when exposed to a concentration of 0.6 mg/mL. Studies when evaluating the embryotoxicity of *Euphorbia milii* latex [30] and aqueous extract of *Moringa oleifera* flower [31] observed toxicity to *B. glabrata* embryos; however, when compared to the preparations of *B. monandra*, they required higher concentrations or exposure time (0.2 mg/mL in 96 h and 0.5 mg/mL in 24 h, respectively) to produce a toxic effect (LC_50_ of 0.03 mg/mL and 2.37 mg/mL, respectively). Table 2 shows the values found for the lethal concentrations LC_10_, LC_50_ and LC_90_ with both preparations.

Although the mechanism of action has not yet been elucidated, our results demonstrate that *B. monandra* preparations have high toxicity and are capable of penetrating both the membrane and the gelatinous mass of the egg containing an embryo and causing unviability, as observed in Figure 3. The initial stages of blastula and gastrula showed a higher susceptibility to our preparations, a result that was similar to that observed with usnic acid [32]. This effect is attributed in the initial stages to the strong cell proliferation observed in embryos in both stages, which make them more subject to teratogenic and lethal effects even in acute exposures [33,34]. Among the malformations observed, developmental delays stand out (Figure 3M), as well as hydropic embryos (Figure 3P) and shell malformations (Figure 3N). According to the study using saline extract and fraction enriched with lectin from *Parkia pendula* seeds [35], the different alterations presented after exposure may be associated with the capacity of protein/lectin to bind to different carbohydrates, causing damage, inhibition of hatching and death of these embryos. Embryos with teratogenic effects rarely hatch [36], thus, the presence of morphological alterations in embryos exposed to *B. monandra* preparations demonstrate the capacity to inhibit development and hatching, consequently causing population control of this host. 

### 2.3. Effect of Preparations on Adult Snails of B. glabrata

**Figure 4:**
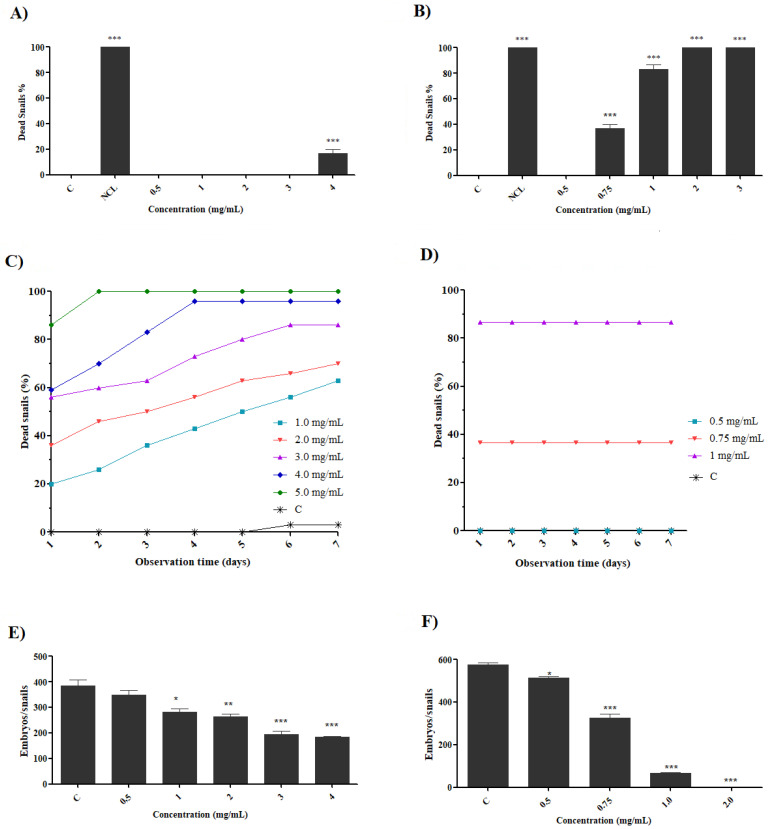
Molluscicidal activity against adult *B. glabrata* snails. Snails exposed to saline extract (**A**) and fraction for 24 h (**B**); snails exposed to saline extract (**C**) and fraction after 7 days of observation (**D**); fecundity of snails exposed to saline extract (**E**) and fraction after 7 days of observation (**F**). C = negative control; NCL = niclosamide: 1.0 µg/mL. Each concentration was compared with the negative control: significance * = *p* < 0.05; ** = *p* < 0.01 and *** = *p* < 0.001.

The exposure of the preparations on adult snails of *B. glabrata* showed a dose-dependent effect, as observed in Figure 4. The saline extract showed a mortality percentage of 16.6% at a concentration of 4.0 mg/mL in 24 h (Figure 4A). However, the fraction was shown to be more active in the concentrations analyzed, with mortality percentages of 83.3% at a concentration of 1.0 mg/mL and 100% at a concentration of 2.0 mg/mL (Figure 4B). When evaluated for 7 days after exposure, the saline extract (48 h) presented increasing mortality (Figure 4C) with percentages of 63% to 100% (0.5–4.0 mg/mL) while the fraction (24 h) was constant (Figure 4D). All *B. glabrata* snails that died during exposure demonstrated intense retraction, mucus production and hemolymph release. Similar alterations, such as tissue damage and loss of biological functions to *B. glabrata* snails, were observed with exposure to the saline extract and fraction of *P. pendula* [35].

The population control of the intermediate host is of paramount importance, because the interruption of the life cycle of the parasite prevents the emergence of new cases of the disease [37]. Thus, the search for plant preparations that present molluscicide activity has increased in recent decades [38,39,40]. Samples containing secondary metabolites (saponins, tannins, steroids and flavonoids) were suggested as the main agents [41,42], thus, secondary metabolites present in the leaves of *B. monandra* indicate constituents capable of presenting molluscicidal action. The molluscicidal activity of chlorophyll extracts were analyzed for *Biomphalaria alexandrina* snails and *Leptodactylus natalensis* and a dose-responsive reduction in survival rates for these vectors was observed [43]. In another trial for the population control of snails (*Pomacea canaliculata*), this reduction was also observed using preparations of *Nerium indicum* leaves, but at a longer exposure time (96 h) [44].

The reduction in fertility and fecundity of snails due to exposure to molluscicide agents can help in population control [37,45]. The preparations tested in the present study induced a decrease in egg oviposition, both in the case of the saline extract (reduction from 10.55% to 52.65% at 0.5 and 4.0 mg/mL, respectively) (Figure 4E) and the fraction (reduction from 9.62% to 88.52% at 0.5–1.0 mg/mL, respectively) (Figure 4F). Effects such as reduced egg hatching and decreased fecundity rates were also seen when evaluating the toxic capacity of *Ramalina aspera* [16], potassium usnate [36] and curcumin [46] on *B. glabrata*. Studies have suggested that the reduction in egg deposition of snails when exposed to *Haplophyllum tuberculatum* [47] and organotin [48] is associated with the decreased biosynthesis of hormones such as progesterone and testosterone, which they associated with decreased fertility.

### 2.4. Effect of Preparations on B. glabrata Hemocytes

**Figure 5:**
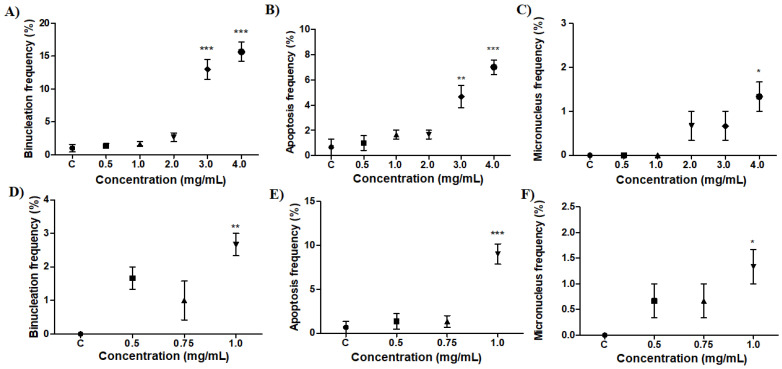
Morphological changes after exposure to different concentrations of saline extract and fraction. Binuclear cells (**A**), apoptosis (**B**) and micronucleus alterations after exposure to saline extract (**C**); binuclear cells (**D**), apoptosis (**E**) and micronucleus alterations after exposure to the fraction (**F**). C: negative control. Dot shapes are only to differentiate concentrations. Each concentration was compared with the negative control: significance * = *p* < 0.05; ** = *p* < 0.01 and *** = *p* < 0.001.

As observed in Figure 5, *B. monandra* preparations showed morphological alterations in hemocytes, such as binuclear cells (BN), micronucleus (MN) and cells with morphological characteristics of apoptosis (PA) with reduced cytoplasm and nuclear fragmentation. These typical features of apoptosis are also described when the toxicity of cadmium telluride (CdTe) quantum dots suspensions against *B. glabrata* was evaluated [49]. 

The negative control presented hyalinocytes and granulocytes, as well as a few morphological alterations, such as BN and AP. The saline extract had a significant effect for BN (Figure 5A) and AP (Figure 5B) at the highest concentrations (3.0 and 4.0 mg/mL) and MN (Figure 5C) only at 4.0 mg/mL when compared to the negative control group, with ± mean standard deviation of 13 ± 2.6 and 15.6 ± 2.5 (BN); 4.6 ± 1.5 and 7 ± 1 (AP) and 1.3 ± 0.5 (MN), respectively. However, for the fraction, the cellular alterations observed were AP (Figure 5E), BN (Figure 5D) and MN (Figure 5F) only at 1.0 mg/mL with significant differences with mean ± standard deviation of 9 ± 2, 2.6 ± 0.5 and 1.3 ± 0.5, respectively, in relation to the negative control group. 

Hemocytes are indispensable in the immunological response of snails and their increase may be related to several factors, such as environmental changes and exposure to pathogens and/or pollutants, making it possible to perceive and quantify changes observed in them [50,51,52,53]. Apoptosis is a self-induced mechanism of death and an important physiological process for homeostasis and immune response [54]. Similar results are presented with potassium usnate [36] and extract and fraction of *P. pendula* [35], demonstrating the importance of the study with AP and BN cells to evaluate the toxic effects on *B. glabrata* hemocytes. Thus, the preparations showed significant cytotoxic effects in the highest concentrations evaluated. However, it is possible to observe these cellular alterations in most concentrations used, which demonstrates the toxic character of *B. monandra* preparations.

### 2.5. Comet Test

**Figure 6:**
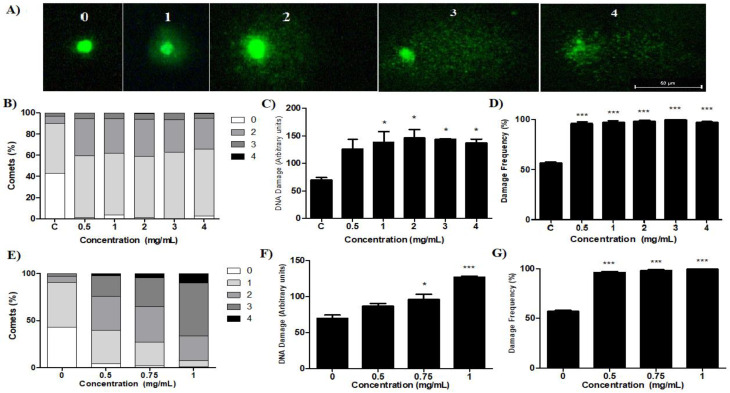
Comet assay images obtained from *B. glabrata* hemocytes stained with Sybrsafe™, and DNA damage after exposure to different concentrations of saline extract and fraction. Different degrees of damage, where 0 indicates absence and 1 to 4 indicates increasing DNA damage (**A**), damage levels (**B**), index (**C**) and frequency of damage after exposure to saline extract (**D**); damage levels (**E**), index (**F**) and damage frequency after exposure to fraction (**G**). C: negative control. * *p* < 0.05 and *** *p* < 0.001.

Figure 6A shows the DNA alterations that were quantified and classified into the five categories of damage. 

Snails exposed to the saline extract showed different levels of damage category (Figure 6B) according to concentrations of 0.5 to 4.0 mg/mL, with levels 1 (58.3–63.3%) and 2 (35–29%), respectively. As for the index (Figure 6C) and frequency of damage (Figure 6D), significant differences were observed in all concentrations with index values 135 to 146 and percentages from 96% to 100%, respectively, for the concentrations used. The fraction showed an increase in DNA changes (Figure 6E) at the highest concentration (1.0 mg/mL), levels 2 (26%), 3 (56%) and 4 (10.33%). The index (Figure 6F) and the frequency of damage (Figure 6G) showed significance at concentrations of 0.75 and 1.0 mg/mL, with values of 95.81% and 98%, and 127.0 and 99.3%, respectively. Studies highlight the sensitivity that the comet assay must assess DNA damage through the electrophoretic migration of nucleotides present in lysed cells in a thin layer of agarose [55,56]. Although the mechanisms through which our preparations interact with the cell producing DNA damage are not elucidated, we can state that both preparations produce genotoxic damage in *B. glabrata* hemocytes. However, studies suggest that oxygen reactive species, produced due to the oxidation of the protoporphyrinogen oxidase enzyme, may induce genotoxicity, damaging the DNA chain and modifying its nitrogen bases [57,58]. Furthermore, other studies also suggest that damage to DNA and other macromolecules may result from superoxide, hydrogen peroxide and hydroxyl radicals that result from cellular metabolism [59]. Thus, we can suggest that the various rates of DNA damage seen with our preparations may be corroborating oxidative stress, which in an uncontrolled way results in a breakdown of the DNA tape [35,60]. Studies also reported an increase in DNA damage rates in groups treated with household slat [61], sodium selenium [62] and aqueous extract of *Anagallis arvensis* [63], which corroborates our results.

### 2.6. Evaluation of Toxicity in S. mansoni Cercariae

The parameters of motility and mortality of the cercariae exposed to the preparations are observed in Table 3.

**Figure 7:**
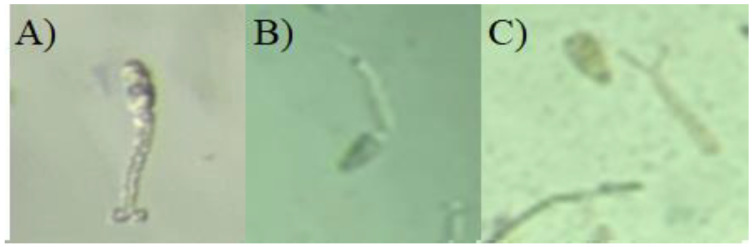
Cercariae of *S. mansoni* exposed to the fraction. Exposed to the negative control (**A**); to niclosamide: 1.0 μg/mL (**B**) and to the fraction with tail and cercarial body separation (**C**).

Figure 7 presents the different exposures: (A) to the negative control presenting maintenance of the cercariae structure, (B) to niclosamide with total death of the cercariae and (C) to the fraction, with separation of tail and cercariae body.

After 60 min of exposure to the saline extract, it is possible to observe reduction in movement at 3.0 mg/mL with a motility score of 1 (presenting contortions and movement in only one extremity). The fraction, in addition to a reduction in motility (1.0 mg/mL), presented total mortality of the cercariae, at a concentration of 2.0 mg/mL for 90 min, demonstrating to be more toxic. Studies reveal that this effect on the motility of the cercariae occurs because the catalytic activity of the neurotransmitter acetylcholinesterase is reduced, due to damage to the parasympathetic nervous system level of the cercariae [64,65], and observed changes in the motility of *S. mansoni* cercariae when exposed 120 min to the aqueous extract of *Glinus lotoides* [39] that resulted in low infectivity, and, consequently, a reduction in parasitic load. When the cercariae’s mortality was evaluated in exposure to essential oil (EO) of *Croton rudolphianus* leaf [40] and divaricatic acid [12], respectively, a reduction in dose-dependent motility related to exposure time could also be observed. In addition to these evaluations, the reduction in motility and mortality came through exposure rough ether extract of *Ramalima* *aspera* [16] and potassium usnate [65].

### 2.7. Artemia Salina Acute Test

**Figure 8:**
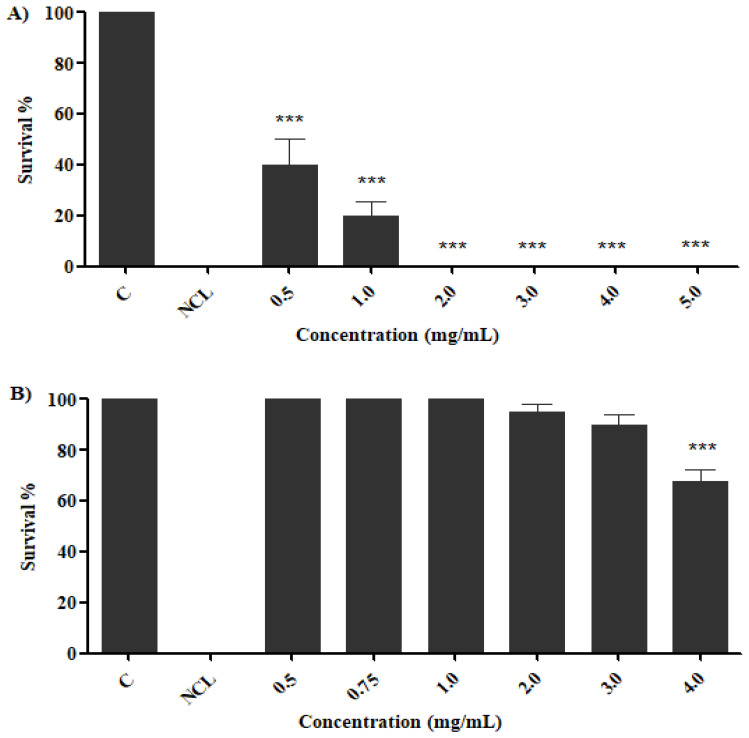
Survival of *A. salina* to *B. monandra* preparations after 24 h exposure. Exposed to saline extract (**A**) and fraction (**B**). C: negative control; NCL: niclosamide: 1.0 µg/mL. Each concentration was compared with negative control: significance *** = *p* < 0.001.

The potential ecotoxicity of the preparations was assessed with the crustacean *A. salina* (Figure 8).

The extract revealed toxicity in the concentrations used (0.5–5.0 mg/mL) with survival percentages of 50–0% (Figure 8A), while the fraction, at concentrations of 0.5 mg/mL to 3.0 mg/mL, revealed 100% to 90% survival (Figure 8B). Thus, when compared to niclosamide (1 μg/mL), the preparations revealed higher survival of *A. salina* nauplii, especially to the fraction, even when evaluated under molluscicide concentrations. This evaluation is necessary, because the use of molluscicides usually results in environmental toxicity, affecting species that inhabit the target locality, thus making the use punctual and restricted, as well as the affected aquatic species being constantly monitored [66].

## 3. Materials and Methods

### 3.1. Plant

Leaves of *B. monandra* were collected at the Academic Center of Vitória (CAV) in Alto José Leal, Vitória de Santo Antão (8°06′59.5″ S 35°17′55.1″ W), Pernambuco, Brazil.

### 3.2. Animals

Juveniles of adult snails of *B. glabrata* were collected in the municipality of São Lourenço da Mata (Pernambuco, Brazil) and kept in the aquaria of the Radiobiology Laboratory of the Department of Biophysics and Radiobiology of UFPE and used as experimental animals. The snails were kept in aquariums with chlorinated filtered water (temperature: 25 ± 3 °C, pH 7.0) with light/dark cycle (12 h) and fed ad libitum daily with lettuce leaves (*Lactuca sativa*).

### 3.3. Preparation of Saline Extract and Fraction of B. monandra

To obtain the saline extract, the collected leaves were washed in running water followed by distilled water and allowed to dry at room temperature for 2 days. The dried leaves were sprayed in a multiprocessor of extracts (10%, *w*/*v*) and the powder (15 g) was added to 150 mL of citrate-phosphate buffer 10 mM, pH 6.5, containing NaCl 0.15 M. The mixture was maintained overnight at 4 °C under gentle agitation using a magnetic stirrer. For the fraction, after the execution of the previous step, the material was passed through gauze and centrifuged for 15 min at 12,000 *g* at 4 °C. Ammonium sulfate with 60% saturation was added to the supernatant to precipitate proteins; the material was kept under gentle agitation at 28 °C for 4 h, then centrifuged (12,000 *g*, 4 °C, 15 min) and lyophilized.

### 3.4. Protein Concentration and Hemagglutinating Activity

The protein concentration in the preparations was determined using bovine serum albumin as standard (0.05–05 μg/mL) [67]. Hemagglutinating activity (HA) was used to determine the presence of lectin [68]. The hemagglutination unit was defined as a value related to the highest dilution of the sample that agglutinated the erythrocytes and the specific HA (SHA), the HA value divided by the protein concentration value.

### 3.5. Phytochemical Screening

The saline extract and the fraction were analyzed for the presence of secondary metabolites according to standard procedures, using different reagents as shown in Table 4.

Samples and reference compounds were manually applied to chromatographic plates of silica gel 60 F254 (Macherey-Nagel GmbH & Co. KG^®^ (Düren, Germany). The plates were developed in vats after saturation with the mobile phase. The tank was saturated for 15 min at room temperature and the bands were applied. The samples were applied to 0.5 cm from the origin and with 0.5 cm ending from the end of the plate. After plate elution, they were dried at room temperature and observed under ultraviolet light of 254 and 365 nm and visible light. Next, they were revealed with specific reagents for each metabolite and the bands obtained were compared to the corresponding pattern. The order of application and visualization followed pattern, extract and fraction, while for the detection of lectin, the electrophoresis profile in polyacrylamide gel under denaturing conditions was observed [69]. The standard protein levels used on this research were phosphorylase b (97 KDa), bovine serum albumin (66 KDa), ovalbumin (45 KDa), carbonic anhydrase (30 KDa), trypsin inhibitor (20.1 KDa) and α-lactoalbumin (14.4 KDa), acquired by GE Healthcare (Chicago, IL, USA). The gels were colored with bright Coomassie blue G 250, with the following order of application: fraction, extract and lectin BmoLL.

### 3.6. High Performance Liquid Chromatography (HPLC) Analysis

HPLC analysis was developed in a HPLC Ultimate 3000 (Thermo Fisher Scientific, Waltham, MA, USA) system, coupled to a photodiode arrangement detector (DAD; Thermo Fisher Scientific) and equipped with binary pump (HPG-3x00RS, Thermo Fisher Scientific), degassing and automatic sampler equipped with a 20 μL loop (ACC-3000, Thermo Fisher Scientific). The wavelengths of analysis were 270 and 350 nm. Chromatographic dissections were obtained with a C_18_ column (250 mm × 4.6 mm d.i., 5 μm) Supelco^®^ equipped with pre-column (C_18_ of 4 mm × 3.9 mm; Phenomenex^®^). The two-way sections were performed at a temperature of 26 ± 1 °C. The mobile phase consisted of ultrapure water (A) and methanol (B), both acidified with trifluoroacetic acid at 0.05% and flow of 0.8 mL/min. A gradient program was applied as follows: 0–10 min, 5–20% B; 10–14 min, 20–25% B; 14–18 min, 25–40% B; 18–25 min, 40–80% B; 25–30 min, 80% B; 30–34 min, 80–5% B; 34–36 min, 5% B. Data were analyzed after triplicate injection and processed using Chromeleon software (Dionex/Thermo Fisher Scientific, Waltham, MA, USA). Additionally, the content of rutin was calculated based on the equation of the calibration curve obtained for the rutin standard (>94%, Sigma-Aldrich^®^, St. Louis, MO, USA). The calibration curve was obtained with 5 points, in the concentration range of 2.00–3.00 µg/mL, and evaluated in triplicate of each concentration. The R2 value was greater than 0.99 (=0.9953). The curve obtained is included in the supplementary material.

### 3.7. Toxicity of Preparations in Embryonic Stages of B. glabrata

The embryos were collected from aquariums containing *B. glabrata* snails and separated (n = 100) in the stages of blastula (0 to 15 h), gastrula (24 to 39 h), trochophore (48 to 87 h), veliger (96 to 111 h) and hippo stage (144 to 168 h) and exposed in Petri dishes for 24 h preparations at different concentrations (0.0125, 0.025, 0.05, 0.1, 0.2, 0.4 and 0.6 mg/mL). For the negative and positive control, dechlorinated filtered water and niclosamide (1 μg/mL) were used, respectively. After exposure, the embryos were washed (dechlorinated filtered water), transferred and kept in plates containing chlorinated filtered water, monitored for 7 days, and classified as viable (normal) and unviable (malformations and dead) [65]. Two independent experiments were carried out in triplicate.

### 3.8. Toxicity, Fertility and Fecundity Analysis in Adult Snails of B. glabrata

Adult snails (n = 10) with 10–14 mm shell diameter were exposed to *B. monandra* preparations for 24 h, at different concentrations (0.5, 1.0, 2.0, 3.0, 4.0 mg/mL), with a final volume of 60 mL. After exposure, the snails were washed (filtered and dechlorinated water) and observed for 7 days, and the mortality rate recorded daily. The snails were considered dead when they presented no movement, release of hemolymph, retraction and/or discoloration of the shell. The negative and positive control groups were performed under the same conditions although chlorinated filtered water and niclosamide (1 μg/mL) were used, respectively [70]. Two independent experiments were carried out in triplicate.

The effect on fertility and fecundity was performed by the analysis of embryos deposited by the surviving snails after exposure of the preparations at different concentrations (0.5–4.0 mg/mL). The egg position of the embryos was recorded for seven consecutive days, started after 24 h of exposure, being analyzed until complete hatching in the negative control group (dechlorinated filtered water). Then, after hatching, the embryos were counted and classified as viable (normal) and unfeasible (malformed and dead) [36].

### 3.9. Evaluation of Cytotoxicity in B. glabrata Snail Hemocytes

Adult snails (n = 10) were exposed to the sublethal concentrations of saline extract (0.5–4.0 mg/mL) and fraction (0.5–1.0 mg/mL) for 24 h. After exposure, the snails were placed in containers with chlorinated filtered water for another 24 h. Negative control was used under the same conditions only with dechlorinated filtered water. Surviving mollusks (three per concentration) were randomly selected for hemolymph collection and morphological analysis [55,71]. Hemolymph (100 μL) was collected and deposited in microscopic slides and then ethylenediaminetetraacetic acid (EDTA) in Ringer’s solution (10 mM) was added and stored in a wet chamber (40 min). The cells were fixed with glutaraldehyde (200 μL; 10 min) in Ringer’s solution at 1% (*v*/*v*) and then washed with Ringer’s solution and flushed with Giemsa at 5% (5 min). Hemocytes (n = 1000 cells/group) were analyzed under an optical microscope (Leica DM1000, Avantor^®^, Radnor Township, PE, USA), with a 100× objective in triplicate.

### 3.10. Evaluation of Genotoxicity by Comet Assay

Hemolymph (100 μL) collected from *B. glabrata* exposed to sublethal concentrations of saline extract (0.5–431 4.0 mg/mL), fraction (0.5–1.0 mg/mL) or dechlorinated filtered water were homogenized in low melting point agarose (1:1). Immediately, this homogenate was placed on microscope slides, previously covered with a normal melting point agarose layer (1.5%) dissolved in PBS (pH 7.4), kept with coverslip and maintained at 4 °C (10 min). After solidification, the coverslips were removed and the slides incubated in lysis solution (2.5 M NaCl, 100 mM EDTA, 10 mM Tris, 1% Triton-X 100 and 10% DMSO, pH 10.0) for 12 h (4 °C). After lysis, the slides were placed in a horizontal electrophoresis vat containing a buffer and alkaline solution, pH 13.0 (1 mM EDTA and 300 mM NaOH), for 20 min. Subsequently, electrophoresis was started for 20 min (4 °C) at 0.74 V/cm and 300 mA. At the end, the slides were neutralized with Tris-HCl 0.4 M buffer (pH 7.5; 15 min) fixed in absolute alcohol (10 min), dried at room temperature, stored at 4 °C and subsequently analyzed [72].

### 3.11. Fluorescence Microscopy Analysis

The slides were analyzed using a fluorescence microscope (Nikon H550L, Nikon Instruments Inc., Tokyo, Japan) with magnification of 400x, excitation filter: 450–490 nm, emission filter: 500–550 nm and barrier filter: 495 nm. To blush the slides, a SYBR (Invitrogen) solution (50 μL; 1:500) was used and damage to the DNA of the hemocytes was visually evaluated [73]. One hundred nuclei were counted for each replica, totaling 400 nuclei, then classified into 5 categories of DNA damage (0, 1, 2, 3 and 4), depending on the extent of the damage. Category 0 indicates that no damage has occurred, while categories 1 to 4 indicate increasing levels of damage to the genetic material. To assess the degree of DNA damage, the damage index (ID) and the frequency of damage (FD%) were used as parameters. The ID was calculated according to the formula: ID = 0 (number of comets category 0) + 1 (number of comets category 1) + 2 (number of comets category 2) + 3 (number of comets category 3) + 4 (number of comets category 4). The FD% was calculated as the percentage value of all comets with DNA damage (category 1–4) in relation to the total number of comets (categories 0–4).

### 3.12. Activity in S. mansoni Cercariae

Adult snails of *B. glabrata* infected with *S. mansoni* (strain, Belo Horizonte, Brazil) were placed in Becker (60 mL), submerged in filtered and dechlorinated water (100 mL) and exposed to artificial light (60 W; 2 h) to eliminate cercariae. A suspension of approximately 100 cercariae were kept in watch glass and exposed to preparations at different concentrations (0.5–5.0 mg/mL). As negative and positive controls, dechlorinated filtered water and niclosamide (1.0 μg/mL) were used, respectively. The test was performed in triplicate and the lethality of the cercariae was observed at intervals of 15, 30, 60 and 120 min after exposure to preparations with the aid of a stereomicroscope (Model 5289, Lumen, Monroe, LO, USA) [16]. During the intervals, the cercariae were evaluated regarding the motility scores: intermittent tail-first natatory movements (3), reduction in slow-paced movement or on the axis itself (2), lethargy and/or contortions and movement in only one end (1) and complete absence of movement of the cercariae (0).

### 3.13. Environmental Ecotoxicity Assessment Using A. salina

Encysted eggs of *A. salina* (Brine Shrimp Co., Mackay Marine^®^, Great Salt Lake, UT, USA) were placed in a beaker with 500 mL of seawater and constant arenization at room temperature (25 ± 3 °C) for 48 h. After hatching, larvae at Instar II–III stages were collected and divided into experimental groups (n = 10) with the help of a stereomicroscope (Wild M3B, Heerbrugg, Switzerland) as follows: negative controls (seawater), positive control (1.0 μg/mL-niclosamide) and preparations at different concentrations (0.5–5.0 mg/mL) for 24 h to 25 ± 3 °C [74]. Two experiments were carried out in quadruplicate and the evaluation of mortality and survival performed with the help of a stereomicroscope.

### 3.14. Statistical Analysis

Statistical analysis was performed using the software GraphPad Prism 5.0 (San Diego, CA, USA). The analysis of variance (ANOVA) was employed, and the results were expressed as mean ± standard deviation (SD). Tukey’s test was used to identify the difference between groups. The lethal concentrations (LC) required to kill 10%, 50% and 90% of *B. glabrata* (embryos and adult snails) were calculated by Probit analysis with a confidence interval of 95% using the StatPlus^®^ 2009 software (Soft Analyst, Vancouver, BC, Canada). 

## 4. Conclusions

The saline extract and fraction of *B. monandra* are promising candidates for the control of *S. mansoni* infection, since both have had molluscicidal effects against *B. glabrata* and are cercaricidal, along with having low environmental toxicity compared to that of the commercial molluscicide niclosamide. The use of *B. monandra* as a new molluscicide depends on future research to prove its action and environmental safety in large-scale field conditions.

## Figures and Tables

**Table 1:** Unviability of the different embryonic stages of *B. glabrata* exposed to saline extract and fraction of *B. monandra*.

Experimental Groups	Unviable by Test %				
	Embryonic Stages				
	Blastula	Gastrula	Trochophore	Veliger	Hippo Stage
**H_2_O**	1.3 ± 0.5	0.3 ± 0.5	1 ± 1	1 ± 1	1.6 ± 0.6
**NCL**	100 ± 0.0	100 ± 0.0	100 ± 0.0	100 ± 0.0	100 ± 0.0
**Saline Extract (mg/mL)**	**Blastula**	**Gastrula**	**Trochophore**	**Veliger**	**Hippo Stage**
0.0125	25.3 ± 7.0 ^b^	14.0 ± 3.4 ^a^	0.6 ± 0.5	3.6 ± 1.5	1.6 ± 2.0
0.025	33.7 ± 2.0 ^c^	37.0 ± 13.6 ^c^	13.6 ± 6.4 ^c^	11.0 ± 2.0	1.3 ± 0.5
0.05	61.0 ± 12.0 ^c^	55.0 ± 5.3 ^c^	29.6 ± 3.5 ^c^	14.6 ± 4.6 ^b^	5.3 ± 2.0
0.1	87.7 ± 1.5 ^c^	71.3 ± 2.5 ^c^	63.0 ± 4.0 ^c^	23.6 ± 2.0 ^c^	16.6 ± 2.0 ^a^
0.2	100 ± 0.0 ^c^	100 ± 0.0 ^c^	77.3 ± 4.6 ^c^	26.0 ± 5.2 ^c^	33.0 ± 5.2 ^c^
0.4	100 ± 0.0 ^c^	100 ± 0.0 ^c^	90.3 ± 1.1 ^c^	59.3 ± 6.6 ^c^	51.6 ± 17.6 ^c^
0.6	100 ± 0.0 ^c^	100 ± 0.0 ^c^	100 ± 0.0 ^c^	100 ± 0.0 ^c^	100 ± 0.0 ^c^
**Fraction (mg/mL)**	**Blastula**	**Gastrula**	**Trochophore**	**Veliger**	**Hippo Stage**
0.0125	13.3 ± 3.5	4.0 ± 1.0	5.6 ± 3.5	1.3 ± 0.6	2.0 ± 1.0
0.025	21.3 ± 2.5 ^c^	33.0 ± 4.5 ^c^	7.6 ± 2.3	11.3 ± 2.5	3.6 ± 2.5
0.05	37.6 ± 1.5 ^c^	57.6 ± 4.1 ^c^	24.3 ± 3.7 ^c^	29.3 ± 7.2 ^c^	24.3 ± 6.0 ^c^
0.1	65.3 ± 9.0 ^c^	88.6 ± 2.0 ^c^	42.6 ± 3.8 ^c^	51.3 ± 5.5 ^c^	51.0 ± 9.1 ^c^
0.2	100 ± 0.0 ^c^	100 ± 0.0 ^c^	64.0 ± 2.6 ^c^	67.7 ± 4.2 ^c^	57.3 ± 4.7 ^c^
0.4	100 ± 0.0 ^c^	100 ± 0.0 ^c^	95.7 ± 2.5 ^c^	92.7 ± 3.0 ^c^	75.3 ± 4.5 ^c^
0.6	100 ± 0.0 ^c^	100 ± 0.0 ^c^	100 ± 0.0 ^c^	100 ± 0.0 ^c^	100 ± 0.0 ^c^

Values were expressed as mean ± standard deviation. H_2_O = negative control (dechlorinated filtered water); NCL = niclosamide—1.0 µg/mL and different concentrations of extract and fraction of *B. monandra* leaves (mg/mL). Each concentration was compared with the negative control: significance ^a^ = *p* < 0.05; ^b^ = *p* < 0.005; ^c^ = *p* < 0.0001.

**Table 2:** Lethal concentrations of the different embryonic stages of *B. glabrata* after exposure to saline extract and fraction of *B. monandra*.

Lethal Concentrations (LC) (mg/mL)			
**Saline Extract**	**LC_10_**	**LC_50_**	**LC_90_**
**Blastula**	0.0056 (0.0046–0.0067)	0.0420 (0.040–0.0434)	0.1552 (0.1538–0.1567)
**Gastrula**	0.0065 (0.0054–0.0077)	0.0417 (0.0405–0.0428)	0.1613 (0.1601–0.1624)
**Trochophore**	0.0166 (0.0150–0.0183)	0.0897 (0.0880–0.0913)	0.3981 (0.3964–0.3997)
**Veliger**	0.0312 (0.0219–0.0405)	0.3734 (0.3641–0.3827)	0.573 (0.5636–0.5823)
**Hippo Stage**	0.0476 (0.0371–0.0580)	0.3970 (0.3866–0.4075)	0.5925 (0.582–0.6029)
**Adult snails (24 h)**	3.73 (3.15–4.30)	6.6 (6.02–7.18)	9.47 (8.898–10.05)
**Fraction**	**LC_10_**	**LC_50_**	**LC_90_**
**Blastula**	0.0073 (0.005–0.0096)	0.0478 (0.0455–0.0501)	0.1681 (0.1658–0.1703)
**Gastrula**	0.0071 (0.006–0.0082)	0.0419 (0.0408–0.0430)	0.1579 (0.1567–0.1590)
**Trochophore**	0.0227 (0.019–0.0263)	0.1582 (0.1544–0.1620)	0.3791 (0.3753–0.3829)
**Veliger**	0.016 (0.0126–0.0194)	0.0974 (0.094–0.1008)	0.3685 (0.3651–0.3719)
**Hippo Stage**	0.0178 (0.0143–0.0213)	0.0970 (0.0935–0.1005)	0.5489 (0.5454–0.5524)
**Adult Snails (24 h)**	0.37 (0.26–0.49)	0.87 (0.75–0.99)	1.70 (1.58–1.82)

()—95% confidence interval.

**Table 3:** Scores of cercariae exposed to saline extract and fraction of *B. monandra* in relation to exposure time.

Experimental Group (mg/mL)	Exposure Time (Minutes)				
	15 min	30 min	60 min	90 min	120 min
**Controls**					
**Negative Control H_2_O**	3	3	3	3	3
**Niclosamide 0.001**	0	0	0	0	0
***B. monandra*** **Saline extract**					
4.0	3	2	1	1	1
3.0	3	2	1	1	1
2.0	3	3	3	3	3
1.0	3	3	3	3	3
0.5	3	3	3	3	3
***B. monandra*** **Fraction**					
2.0	3	2	1	0	0
1.0	3	2	2	1	1
0.75	3	3	3	2	1
0.5	3	3	3	3	3

Motility score: 3: intermittent *tail-first* natatory movements. 2: Reduction in movement with slow pace. 1: Contortions and movement at only one end. 0: Complete absence of movement/death.

**Table 4:** Chromatographic conditions for identification of chemical profile by thin layer chromatography (TLC).

Metabolite Class	System	Reference Compounds	Developer
Cinnamic derivatives	(90:5:5)	Caffeic acid	AlCl_3_
Flavonoids	(90:5:5)	Quercetin	AlCl_3_
Hydrolysable tannins	(90:5:5)	Gallic acid	FeCl_3_
Condensed Tannins	(90:5:5)	Catechin	Vanillin Hydrochloric + Δ
Coumarin	(50:50:50)	Coumarin	KOH
Terpenes/Steroids	(90:10)	β-sitosterol	Liebermann-Burchard + Δ
Saponins	(16:10:2.5)	Escin	Liebermann-Burchard + Δ
Anthracenes	(20:30:15:0.5)	Sennoside B	HNO_3_ + Δ + KOH
Sugars	(100:11:11:26)	Glucose	Thymol + H_2_SO_4_ + Δ
Alkaloids	(70:20:10)	Piperine	Dragendorff

AlCl_3_: 5% aluminum chloride in methanol; FeCl_3_: railway; Δ: enthalphy/heating; KOH: potassium hydroxide; HNO_3_: nitric acid; H_2_SO_4_: sulfuric acid. 90:5:5 = ethyl acetate, formic acid and water; 50:50:50 = toluene, ethyl ether and glacial acetic acid (saturation); 90:10 = toluene, ethyl acetate; 100:11:11:26 = ethyl acetate, glacial acetic acid, formic acid and water; 16:10:2.5 = chloroform, methanol and water; 20:30:15:0.5 = ethyl acetate, n-butyl alcohol, water and glacial acetic acid; 70:20:10 = toluene, ethyl acetate, diethylamine.

## Data Availability

Not applicable.

## Supplementary Materials

The following supporting information can be downloaded at: https://www.mdpi.com/article/10.3390/molecules27154993/s1, Figure S1. Calibration curve obtained for standard rutin.

## Sample Availability

Samples of the compounds of *Bauhinia monandra* preparations are available from the authors.
