# Supplementary figures and images for: Effect of Bauhinia monandra Kurz Leaf Preparations on Embryonic Stages and Adult Snails of Biomphalaria glabrata (Say, 1818), Schistosoma mansoni Cercariae and Toxicity in Artemia salina
Source: Molecules. 2022 Aug 5;27(15):4993. doi: 10.3390/molecules27154993 (PMC9370106; doi:10.3390/molecules27154993)

## Supplementary Materials

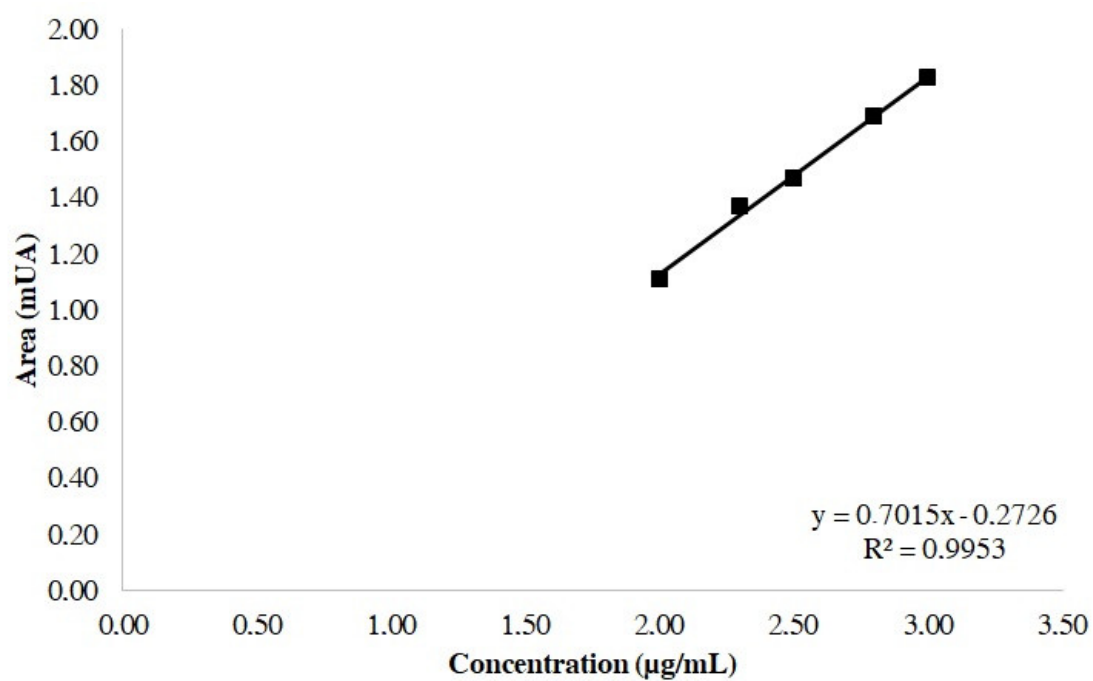

**Figure S1.** Calibration curve obtained for standard rutin.

Supplement: Supplementary file 1 [file molecules-27-04993-s001.zip › molecules-1779963-supplementary.pdf]
